# Organ dysfunction in the resuscitation phase of critical burn patients

**DOI:** 10.1186/cc11069

**Published:** 2012-03-20

**Authors:** A Agrifoglio, M Sánchez, M Hernández, J Camacho, L Cachafeiro, M Asensio, E Herrero, A García de Lorenzo, M Jiménez

**Affiliations:** 1 Hospital Universitario La Paz, Madrid, Spain

## Introduction

Sequential Organ Failure Assessment (SOFA) is useful to assess organ dysfunction in burn patients [1]. The aim of this study was to determine the change in organ dysfunction from admission to day 3.

## Methods

We performed a prospective observational cohort study with critical burn patients (total body surface area (TBSA) >20% and/or inhalation injury) admitted to our burn ICU from September 2008 to December 2010. Epidemiological data and SOFA score at admission (day 0) and days 1, 2 and 3 were collected.

## Results

Sixty-four patients were enrolled (70% men) with mean age of 48.2 ± 19.0 years; Abbreviated Burn Severity Index (ABSI): 8.78 ± 2.59; APACHE II score: 13.5 ± 6.5. Twenty-three patients (35.9%) had inhalation injury and 19 patients (29.7%) died. The SOFA score was increased from day 0 to day 3. At admission the most frequent dysfunctions were cardiovascular and respiratory. The respiratory was similar in the next days and the cardiovascular dysfunction worsened (Table 1). Haematological dysfunction appeared at day 3 (1.05 ± 1.0) and neurological, renal and hepatic dysfunction were uncommon in the resuscitation phase.

**Table 1:** SOFA during the resuscitation phase

	Day 0	Day 1	Day 2	Day 3
SOFA	3.40 ± 2.48	4.26 ± 2.99	4.95 ± 3.04	5.25 ± 3.25
Respiratory	1.38 ± 1.12	1.32 ± 1.09	1.81 ± 1.09	1.76 ± 1.07
Cardiovascular	1.19 ± 1.76	2.06 ± 1.94	2.10 ± 1.85	2.22 ± 1.89

## Conclusion

In the resuscitation phase of our critical burn patients the initial dysfunction was respiratory and cardiovascular, progressing later to cardiovascular dysfunction and haematological dysfunction appearing at the third day of admission. Knowing the possible evolution of organ dysfunction may help early detection and treatment.
